# Fourth finger dependence of high-functioning autism spectrum disorder in multi-digit force coordination

**DOI:** 10.1038/s41598-018-38421-6

**Published:** 2019-02-11

**Authors:** Shunta Togo, Takashi Itahashi, Ryuichiro Hashimoto, Chang Cai, Chieko Kanai, Nobumasa Kato, Hiroshi Imamizu

**Affiliations:** 1 Graduate School of Informatics and Engineering, The University of Electro-Communications, Tokyo, Japan; 2 Brain Science Inspired Life Support Research Center, The University of Electro-Communications, Tokyo, Japan; 3 Cognitive Mechanisms Laboratories, Advanced Telecommunications Research Institute International, Kyoto, Japan; 4 Medical Institute of Developmental Disabilities Research, Showa University, Tokyo, Japan; 5 Department of Psychology, Graduate School of Humanities and Sociology, The University of Tokyo, Tokyo, Japan

## Abstract

A number of studies have reported that the digit ratio 2D:4D (length of the second finger divided by length of the fourth finger) is smaller (longer fourth digit) in autism spectrum disorder (ASD) than in typically developed (TD) controls. Because form and function are closely related in biological systems, we hypothesized that the 4D dominance occurs in not only finger morphology but also physical performance in ASD. Individuals with ASD and TD controls participated in a multi-digit force-producing task. Individuals with ASD showed a significant 4D dependence compared to TD controls in the task. We found a significant correlation between 4D dependence and scores of the standard diagnostic instrument across individuals with ASD. Our analysis of functional connectivity in resting-state functional MRI suggests that connectivity between the visual cortex and the cerebellum contributes to the 4D dependence. Collectively, these results extend the 2D:4D ratio beyond being a morphological marker to being involved in motor functions in the form of 4D dependence in a multi-digit force task.

## Introduction

A number of studies have reported that 2D:4D (length of the second finger divided by length of the fourth finger) is smaller in autism spectrum disorder (ASD) or ASD-affected individuals than in unaffected controls^1,2^. The digit ratio 2D:4D has been proposed as a retrospective marker of a prenatal testosterone^3,4^, and a smaller value (i.e., long ring finger relative to index finger) corresponds to more male-typical characteristics^5,6^. Thus, the small 2D:4D ratio is consistent with the ‘extreme male brain’ theory of autism^7,8^ and suggests that prenatal testosterone drives ASD characteristics in brains with specific backgrounds^1^.

In the current study, we hypothesized that the small 2D:4D ratio is observed in not only finger morphology but also physical performance (motor function) in ASD. Our rationale for this hypothesis is that form and function are closely related in biological systems^9^, suggesting the possibility of 4D dominance in not only morphology but also function. To examine this hypothesis, we adopted a multi-digit force-sharing task to investigate the relationships among physical performances of individual fingers^10–12^. In this task, participants adjusted the total force of their four fingers to a target force, while the relative contribution of each finger-force to the total force is arbitrarily determined by the participants.

We investigated the difference in finger-force-sharing patterns between participants with high-functioning ASD and typically developed (TD) controls. According to our hypothesis, we predict that participants with ASD would rely on the 4D during force adjustment, and that the force ratio of the 4D to the total force of the four fingers would be higher (4D dependence) in participants with ASD than TD controls. We actually found a significant 4D dependence in the force-sharing patterns of participants with ASD. This was found in a specific (visual feedback control) phase of the task. Moreover, we found a significant correlation between the 4D dependence and scores of the standard diagnostic instrument (Autism Diagnostic Observation Schedule: ADOS) across individuals with ASD. Our analysis of functional connectivity in resting-state functional MRI (rs-fMRI) suggested that connectivity between the visual cortex and the cerebellum may contribute to the distinct force-sharing pattern in ASD during the visual feedback control phase.

## Results

### Multi-digit force-sharing task

**Figure 1:**
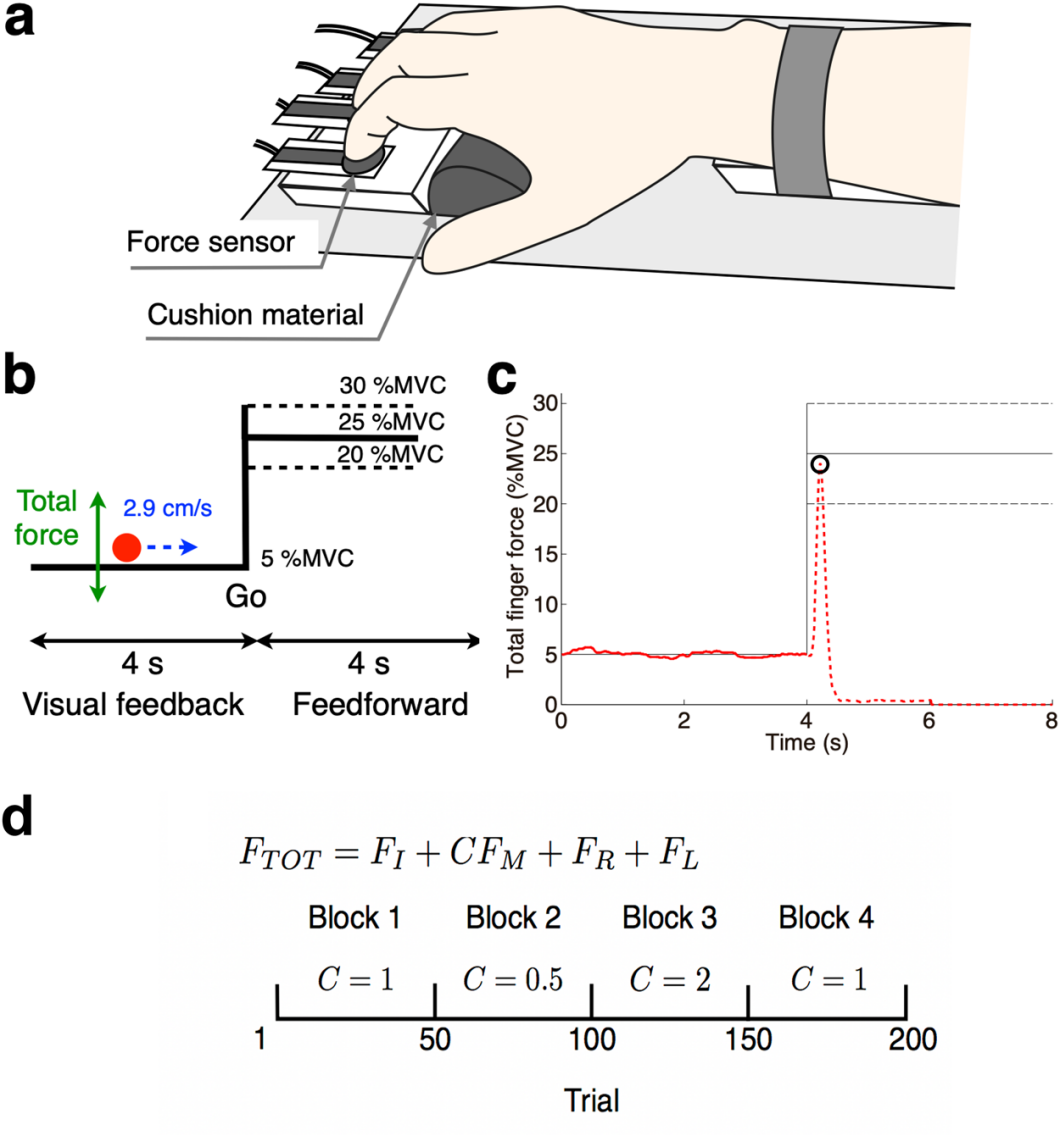
Experimental setup and analysis method. (**a**) Participants placed their fingers on four custom-made force sensors and their palm on a cushion material. (**b**) A screen was displayed to the participants during a multi-digit force-sharing task. A cursor (red circle) moved from left to right. Its horizontal speed was constant, while its vertical position was proportional to the total force generated by the four fingers. The cursor was visible during a visual feedback phase (left half of screen) but disappeared during a feedforward-control phase (right half). (**c**) Typical force trajectory in a participant’s ‘success’ trial. A moving cursor was visible on the solid trajectory but invisible on the broken trajectory. The open circle indicates the force peak at which a stationary cursor appeared at the end of the trial. (**d**) Task schedule of multi-digit force-sharing task. Total finger force *F*_*TOT*_ consists of the finger forces generated by the index (*F*_*I*_), middle (*F*_*M*_), ring (*F*_*R*_), and little (*F*_*L*_) fingers. Middle finger force *F*_*M*_ is weighted by the coefficient *C*. The value of weight coefficient *C* was changed according to the sequence in the bottom panel.

Twenty male individuals with ASD and twenty male TD controls participated in the experiments. Age, handedness, and estimated IQ were matched between the two groups. The main task of the current study was a multi-digit force-sharing task, in which participants placed each of the four fingers (from the second to the fifth digit of the right hand) on a force sensor and exerted isometric force on sensors to control a cursor on a computer screen (Fig. 1a). The cursor moved from left to right at a constant velocity (2.9 cm/s on the screen), and thus the horizontal position (*x*) was determined by the elapsed time from the trial onset independently of the exerted force. By contrast, the vertical position (*y*) was proportional to the total force of the four fingers. Participants were asked to trace a ‘step function-like’ target path with the cursor (Fig. 1b^13^). We gave no instruction on the relative contribution of each finger to the total force, so the fingers’ contributions were arbitrary determined by participants. A trial consisted of a visual feedback-control phase (left half of Fig. 1b) and a feedforward-control phase (right half) as described below.

### Visual feedback-control phase

In the feedback-control phase of each trial, participants were asked to move the cursor along the target path while minimizing the distance between the cursor and the path. The cursor was always visible during this phase. We adjusted the force-cursor relationship before the experiment so that each participant would produce 5% of his maximal voluntary contraction (MVC) of the total finger force when the cursor is on the target path (see Methods). We calculated a tracing error in each trial as a root mean square (RMS) error between the target force (5% MVC) and the total force produced by the participants.

### Feedforward-control phase

In the feedforward-control phase of each trial, participants were asked to produce a quick single pulse of the total force when the cursor arrived at the mid-point vertical line (Fig. 1b and c). By generating a pulse force, they made the cursor jump to the level of the right horizontal solid line, which corresponded to 25% of individual MVC. Because the cursor disappeared after crossing the mid-point line, participants conducted this task without visual feedback (feedforward control). At the end of each trial, a stationary cursor appeared at the peak of the pulse force (open circle in Fig. 1c) to inform participants of their performance. The trial was considered as “success” if the peak of the force was within a range of 25 ± 5% of the MVC, that is, if the stationary cursor appeared in the area between the broken lines (Fig. 1c). Feedback was also given to the participants as a message of “success” or “failure” on the screen. A typical force trajectory from a participant is shown in Fig. 1c.

### Blocks

Each participant performed in four 50-trial blocks. We changed the weight for the third digit (middle finger) across the blocks (Fig. 1d) to investigate the force-sharing patterns under variable conditions. The weight was 1.0 in the first and fourth blocks while it was 0.5 (reduced) and 2.0 (magnified) in the second and third blocks, respectively. Participants were not informed of this change.

### Results of feedback-control phase

#### Tracing error

Regarding the tracing error in the feedback-control phase, we could not find a significant difference between the participants with ASD and TD controls across the four blocks (*t*-test: *p* value, *p* = 0.25; *t* value, *t*_(38)_ = 1.17; effect size, *d* = 0.37). No significant difference was identified even if we applied a two-way ANOVA to the error with factors of participant group and block (group effect, *p* value, *p* = 0.055; *F* value, *F*_(3, 152)_ = 3.74; effect size, *η*^2^ = 0.023; block effect, *p* = 0.16, *F*_(3, 152)_ = 1.74, *η*^2^ = 0.032; interaction, *p* = 0.96, *F*_(3, 152)_ = 0.1, *η*^2^ = 0.0018). Thus, individuals with ASD could accomplish the requirement of the feedback-control task at a comparable level with TD controls.

#### Force-sharing patterns

**Figure 2:**
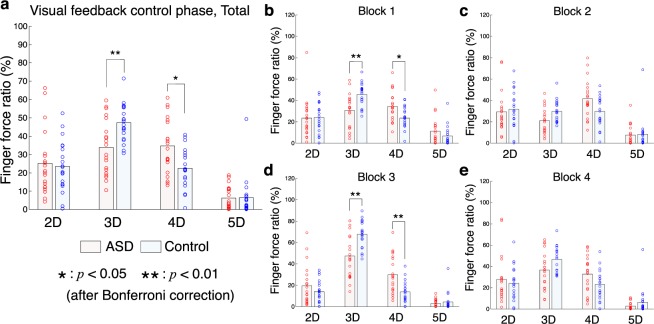
Finger force ratio during visual feedback-control phase. Finger force ratios across all trials (**a**) and finger force ratios from block1 (**b**) to block4 (**e**) are shown. The horizontal axis denotes digits. Light blue and pink bars denote mean values of ASD and control groups, respectively. Circles correspond to individual participants. The finger force of the middle finger (3D) was differently weighted depending on the block. The force ratios were calculated based on the weighted forces. The single and double asterisks denote a significant difference between ASD and control groups with *p* < 0.05 and *p* < 0.01 after Bonferroni correction, respectively.

We compared finger-force-sharing patterns of individuals with ASD to those of TD controls during the feedback-control phase to test our hypothesis that the small 2D:4D ratio (4D dependence) is related to motor performance in ASD. We investigated the force ratio of each finger to the total force across blocks (Fig. 2a) and found a significant interaction effect (participant group × finger) on the force ratio according to a two-way ANOVA (*p* = 0.0001 < 0.01, *F*_(3, 152)_ = 8.01, *η*^2^ = 0.06). This suggests that the force-sharing pattern of individuals with ASD is significantly different from the pattern of TD controls. A post-hoc test indicated that the force ratio of the 4D of participants with ASD was significantly higher than that of TD controls (a stronger 4D dependence: *p* = 0.0228 < 0.05 after Bonferroni correction for four comparisons, *t*_(38)_ = 2.93, *d* = 0.93). By contrast, the force ratio of the third digit of TD controls was significantly higher than that of participants with ASD (*t*-test: *p* = 0.0048 < 0.01 after Bonferroni correction, *t*_(38)_ = 3.52, *d* = 1.11). We analyzed the force ratios separately for blocks (Fig. 2b–e) and confirmed a consistent observation. That is, the force ratio of the 4D was higher in individuals with ASD than TD controls in each of the four blocks, with a statistical significance in the first block (*p* = 0.032 < 0.05 after Bonferroni correction, *t*_(38)_ = 2.79, *d* = 0.88) and the third block (*p* = 0.0044 < 0.01 after Bonferroni correction, *t*_(38)_ = 3.53, *d* = 1.17). These findings suggest that individuals with ASD show a stronger dependence on the fourth digit than TD controls.

#### Correlation analysis between 4D dominance and clinical severity of ASD

**Figure 3:**
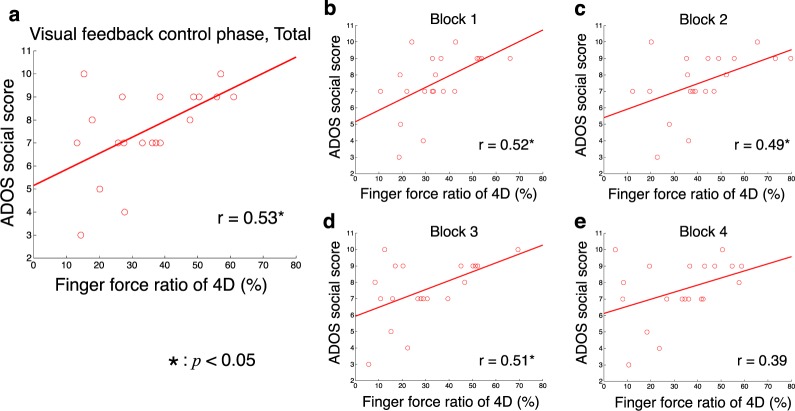
Relationships between force ratio of 4D and ADOS score. The horizontal axis denotes the force ratio of the 4D (ring finger) during visual feedback-control phase across all trials (**a**) and from block1 (**b**) to block4 (**e**). The vertical axis denotes ADOS social score. Circles and lines indicate data of individual participants and linear regressed lines, respectively. Coefficients of correlation (*r*) are presented. The asterisk denotes significant correlation (*p* < 0.05).

We examined the relationship between the ratio of 4D force to total force and the ADOS social score for individuals with ASD. We found a significant positive correlation across blocks (Fig. 3a, *r* = 0.53, *p* = 0.0163 < 0.05). A significant correlation was observed in three of the four blocks when we calculated correlations separately for the blocks (Fig. 3b–e, block 1: *r* = 0.52, *p* = 0.0179 < 0.05; block 2: *r* = 0.49, *p* = 0.0301 < 0.05; block 3: *r* = 0.51, *p* = 0.0209 < 0.05; block 4: *r* = 0.39, *p* = 0.0863). These results suggest a significant correlation between 4D dependence and the degree of ASD symptoms.

### Results of feedforward-control phase

#### Success rate

**Figure 4:**
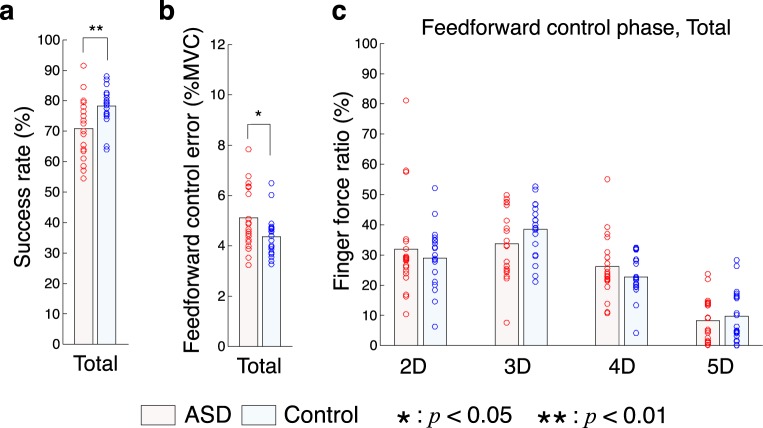
Task results in feedforward control phase. (**a**) Success rate of all participants. Light blue and pink bars denote mean values of ASD and control groups, respectively. Circles show the values of individual participants. (**b**) Aiming error in the feedforward-control phase. (**c**) Finger force ratios across all trials. The horizontal axis denotes digits. Single and double asterisks denote a significant difference between ASD and control groups with *p* < 0.05 and *p* < 0.01, respectively.

Participants with ASD showed a significantly lower success rate (ratio of “success” trials to total trials) than TD controls across blocks (Fig. 4a, *t*-test: *p* = 0.0061 < 0.01, *t*_(38)_ = −2.91, *d* = −0.92). We calculated an aiming error measured by the absolute difference between the target force and the peak of the total force. The aiming error was significantly larger in individuals with ASD than TD controls (Fig. 4b, *t*-test: *p* = 0.032 < 0.05, *t*_(38)_ = 2.23, *d* = 0.70). Data for individual blocks are shown in Fig. S1 These results indicate lower performance of participants with ASD in the feedforward-control phase in comparison to TD controls.

#### Force-sharing patterns

We checked the force-sharing pattern in the feedforward-control phase. Figure 4c shows force ratios of each digit to total force at the time point of the total-force peak. A two-way ANOVA showed significant finger effect (*p* = 4.84 × 10^−31^ < 0.01, *F*_(3, 152)_ = 79.74, *η*^2^ = 0.60) but no significant group effect (*p* = 0.81, *F*_(1, 152)_ = 0.06, *η*^2^ = 0.0001) or interaction (*p* = 0.16, *F*_(3, 152)_ = 1.75, *η*^2^ = 0.013). Therefore, no significant difference in finger-force pattern was identified between participants with ASD and TD controls. This differed from the results during the visual feedback-control phase. Force-sharing patterns in individual blocks are shown in Fig. S2.

### Fundamental properties of the hands

We examined several properties of the hands in both groups as follows.

#### Morphological 2D:4D ratio

We measured the length from the metacarpophalangeal joint to the fingertip for the second and fourth digits and calculated the 2D:4D ratio (left column of Table 1). We could not find a significant difference between the groups, at least for our participants (*p* = 0.24, *t*_(32)_ = 1.21, *d* = 0.42).

**Table 1:** Fundamental properties of the hands in all participants.

	2D:4D ratio	MVC (N)	EN_*I*_	EN_*M*_	EN_R_**	EN_*L*_
ASD	0.958 ± 0.0375	34.00 ± 7.55	0.0125 ± 0.0138	0.0261 ± 0.0303	0.0185 ± 0.0154	0.0686 ± 0.0461
Control	0.943 ± 0.0356	37.52 ± 8.49	0.0340 ± 0.0415	0.0165 ± 0.0219	0.0643 ± 0.0460	0.0499 ± 0.0402

Means and standard deviations (SD) of the morphological second digit to fourth digit (2D:4D) ratio, maximal voluntary contraction (MVC) of total finger force, and enslaving index (EN) are presented. Subscripts denote: *I*, index; *M*, middle; *R*, ring; *L*, little fingers. Double asterisks denote a significant difference between ASD and control groups, *p* < 0.01 after Bonferroni correction.

#### MVC

MVCs of total finger-force were measured before the force-sharing task. Participants were asked to push the four force sensors with their second to fourth digits as strongly as possible. We defined the MVC as the peak of the produced total finger-force (second column of Table 1). We could not find a significant difference in MVC between the groups (*p* = 0.17, *t*_(38)_ = −1.39, *d* = −0.44).

#### Finger enslaving

We investigated the effect of so-called finger enslaving, which may have affected the observed force-sharing pattern. Finger enslaving is the inability of the fingers to exert force fully independent of their neighboring fingers (unintentional force interactions between the fingers). This has been explained by mechanical and neural connections between the muscles controlling the fingers^14^. We asked participants to produce a force only by using a single ‘tested finger’ while measuring forces generated by all four fingers (see Fig. S3). We calculated an enslaving index as the ratio of unintentional forces generated by the fingers other than the tested finger to total force (see Methods). The right four columns in Table 1 show enslaving indices calculated for individual fingers. We applied a two-way ANOVA to the indices and found a significant interaction between the group and finger (*p* = 0.0002, *F*_(3, 152)_ = 7.05, *η*^2^ = 0.1023). This suggests a significant difference in finger-enslaving patterns between the groups. A post-hoc test identified a significantly lower index at the 4D of the individuals with ASD than the TD controls (*t*-test: *p* = 0.0008 < 0.01 after Bonferroni correction, *t*_(38)_ = −4.12, *d* = −1.30). This indicates that participants with ASD could generate the 4D force independently of other fingers.

### Functional connectivity among brain regions

**Figure 5:**
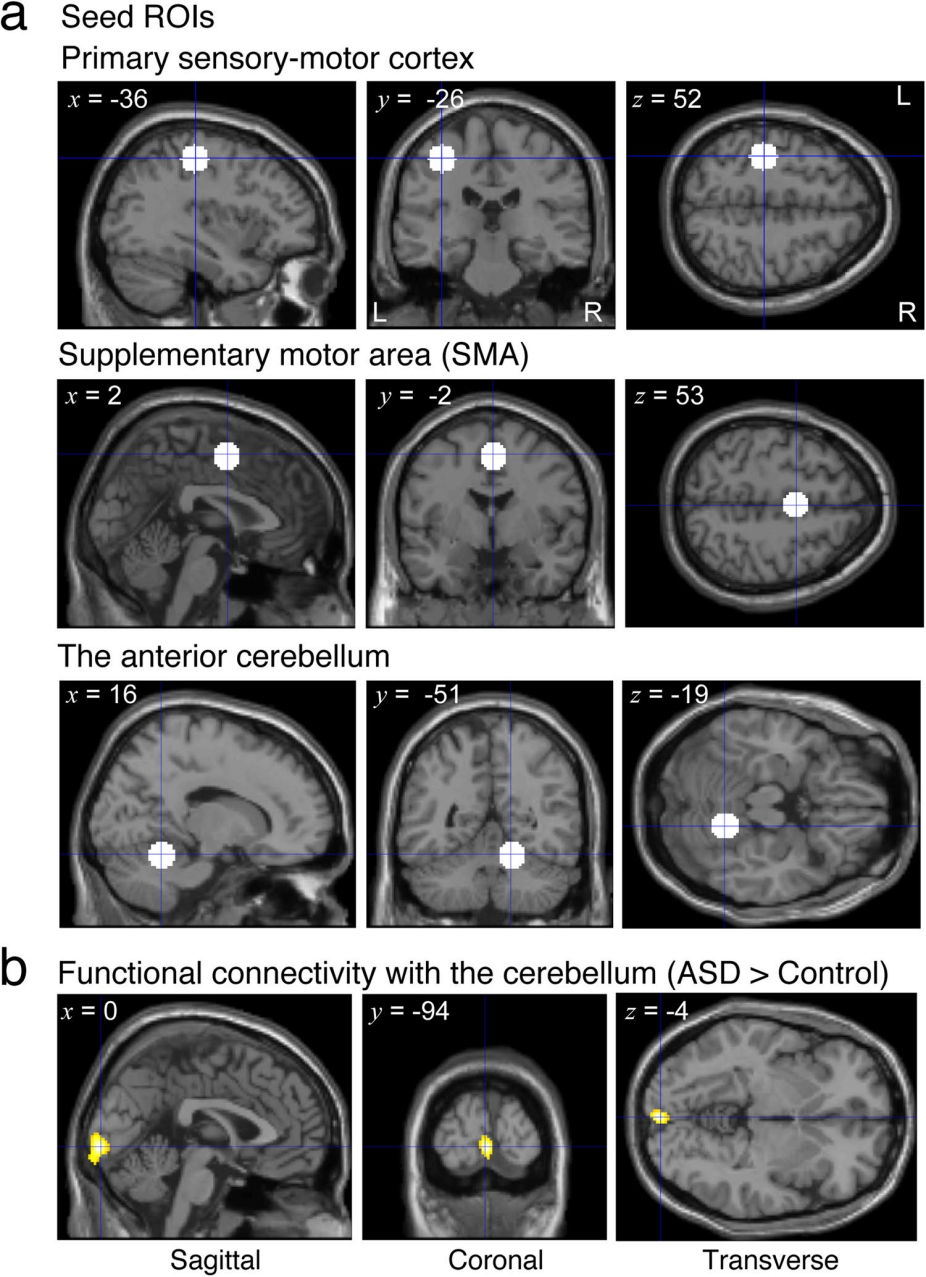
Seed regions and functional connectivity map. (**a**) White circles indicate seed regions (spheres with 7.5-mm radius) in the hand areas of the three brain regions related to control of hand movements. *x*, *y* and *z* represent the center of the spheres in the MNI coordinates. (**b**) Yellow regions indicate a cluster region in the primary visual cortex where functional connectivity with the cerebellum is significantly higher in participants with ASD than TD controls (initial cluster-forming threshold: *P* < 0.001, peak height threshold: *P* < 0.05 FWE corrected). *x*, *y* and *z* represent the peak of correlation value.

We investigated the difference in the resting-state functional connectivity between participants with ASD and TD controls with respect to brain regions related to the control of hand movements. To obtain enough statistical power, we used an rs-fMRI dataset (42 participants with ASD and 43 TD controls) that included 11 participants with ASD and 7 TD controls who performed in the behavioral experiments. We located spherical seed regions (7.5-mm radius) in the “hand areas” of the left primary sensory-motor region, the supplementary motor area (SMA), and the right anterior cerebellum (lobules IV/V) (Fig. 5a). We calculated Pearson’s correlation coefficients in time courses of rs-fMRI activity between these seed regions and every voxel in the brain and then created a while-brain connectivity map for each seed and participant. The maps were contrasted between the groups. As a result, we found a cluster region in the primary visual cortex (Fig. 5b and Table S1) where the functional connectivity with the cerebellar seed was significantly higher for the participants with ASD than TD controls (initial cluster-forming threshold: *p* < 0.001 uncorrected, peak height threshold: *p* < 0.05 family-wise-error [FWE] corrected). This cluster showed significance even when the seed radius was set to 5 mm (*P* = 0.027, FWE corrected) or 10 mm (*p* = 0.006, FWE corrected). Mean correlation value within the cluster was *r* = 0.076 (SD: 0.16) across participants with ASD and *r* = −0.092 (SD: 0.15) across TD controls. We could not find a significantly different functional connectivity between individuals with ASD and TD controls with a seed region in the primary sensory-motor cortex or SMA.

## Discussion

In this study, we tested our hypothesis that 4D dominance is observed in the motor performance of individuals with ASD. On the one hand, participants with ASD showed comparable performance to that of TD controls during the feedback-control phase of a multi-digit force task. When we examined force-sharing patterns during this phase, we found a significant 4D dependence in participants with ASD in comparison to TD controls. Moreover, there was a significantly positive correlation between the degree of 4D dependence and the severity of social interaction deficits as assessed by ADOS (social) for individuals with ASD. On the other hand, participants showed a significantly lower performance (success rate) than TD controls during the feedforward-control phase. We could not find 4D dependence during this phase. Therefore, our results supported the above hypothesis, at least for feedback control of finger forces.

### 4D dependence in motor function

We found a significant 4D dependence in the motor performance (finger-force-sharing pattern during feedback-control phase) in participants with ASD. We investigated the correlation between the morphological 2D:4D ratio and the 4D dependence in the force-sharing pattern across blocks for participants with ASD but could not find a significant correlation (*r* = 0.123, *p* = 0.607). Thus, it is unlikely that morphological 2D:4D (longer 4D) directly contributed to the 4D dependence in the force-sharing patterns. These results suggest that individuals with ASD have 4D dependence in motor function independently of the morphological 2D:4D, at least for their results in this experiment.

Our measurement of finger enslaving also supports the 4D dependence in motor function in individuals with ASD. The low enslaving index of the 4D suggests that individuals with ASD could control the 4D force independently of the other fingers. This accounts for the reason why individuals with ASD rely on the 4D when a subtle adjustment of force was required (around 5% of MVC) during the feedback-control phase.

### Extinction of 4D dependence during feedforward-control phase

We found inferior performances (low success rate and high aiming error) for participants with ASD compared to TD controls when the rapid generation of a pulse force was required during the feedforward-control phase. This is consistent with a previous study that reported deficits in feedforward and rapid force control in individuals with ASD^15^.

However, the reason is unclear so far why the 4D dependence in individuals with ASD was observed during the feedback-control phase but not during the feedforward-control phase. We assume this was caused by the difference in required force-strength between the feedback (5% MVC) and the feedforward (25% MVC) control phases. The 5% MVC is relatively low and gave participants a large degree of freedom regarding the configuration of force pattern across fingers. This allowed the 4D dependence to appear during the feedback-control phase. By contrast, the 25% MVC of the total finger force is relatively high, and the force was required to be quickly generated at once in the feedforward-control phase. Thus, the task required a significant and simultaneous contribution of every finger to the total force. This allowed only a small degree of freedom regarding the configuration of the force pattern (see Fig. S4 and its legend for detailed explanation). Therefore, it was probably difficult for the 4D-dependent pattern to appear during the feedforward-control phase.

### Neural correlates of 4D dependence

Since the 4D dependence was observed during the visual feedback-control phase but not during the feedforward-control phase, there is a possibility that it is related to the neural circuits underlying the visual feedback control loop of individuals with ASD. Our functional connectivity analysis found that the activity of the visual cortex was more highly correlated with the cerebellar activity in the ASD group than in the control group (Fig. 5b and Table S1). This result predicts that the contribution of the cerebellum to motor control is high in the ASD group when visual information is critical for task performance (i.e., under a visual feedback control task). Given the cerebellar contribution to learning movement coordination^16^, we can hypothesize that the 4D dominance originated from the coordination patterns of fingers stored in the cerebellum of individuals with ASD. We need further study to test this hypothesis.

### 4D dependence and disorders of motor coordination in ASD

Clues to understanding 4D dependence may be found in disorders of motor coordination, which have been frequently reported for ASD, and in the independent controllability of the 4D found in our current study (see our finger-enslaving analysis). It has been reported that individuals with ASD often present with a developmental coordination disorder^17–24^. Our multi-digit force-sharing task can be considered a motor coordination task in which participants coordinate forces of individual fingers to adjust the total force to a given value (5% MVC or 25% MVC). However, our participants with ASD showed comparable performance with TD controls, at least during the feedback-control phase. Note that 4D dependence was found during the feedback-control phase. Therefore, we can hypothesize that 4D dependence is a control strategy to compensate for the inferior coordination of movements by utilizing independent controllability of the 4D. This strategy is probably acquired during the course of development. The memory of such a control strategy may be stored in the cerebellum.

### Effects of cognitive competence on 4D dependence

All participants with ASD were considered to be high functioning based on their intelligence quotient (IQ) scores (see Methods). Therefore, a problem in cognitive competence unlikely affected ability of participants with ASD to understand and conduct the force-sharing task. However, we cannot deny the possibility that any cognitive competence that cannot be evaluated by IQ scores, might have affected the 4D dependence. The morphological 2D:4D ratio has been reported in many groups other than ASD, such as in elite athletes^25^ and azoospermic men^26^, whose cognitive competences are unlikely different from those of controls. Therefore, future investigation of the force-sharing patterns in such groups would avoid the problem of matching the cognitive competence between participant groups.

### Failure to identify small morphological 2D:4D ratios in participants with ASD

We did not obtain a significant difference in the morphological (physical length) 2D:4D ratio between individuals with ASD and TD controls (Table 1). Possible reasons for this failure are as follows. First, the number of participants in the behavioral experiment was low (*n* = 20 for each group). A meta-analysis study^6^ confirmed the reliability of a low 2D:4D ratio in ASD across different research groups and measurement methods. However, the meta-analysis estimated that the 2D:4D ratio is lowered by approximately 0.5–0.6 SD in individuals with ASD in comparison to TD controls. Based on this estimation, the same study pointed out that a difference of approximately 0.5 SD indicates that most individuals with ASD have a 2D:4D ratio within the normal (TD) range. Therefore, it is not surprising that no statistically significant difference could be identified in our study. Second, our measurement of finger length may not have been accurate enough to capture the subtle difference in 2D:4D ratio. X-rays are required to accurately measure the position of the metacarpophalangeal joint, but we were unable to access an X-ray device. Instead, we carefully measured the finger length by using digital photos of participants’ palms. To evaluate accurate relationship between morphological 2D:4D ratio and the 4D dependence in the force-sharing paradigm, finger length measurements using X-ray imaging is required in future work.

## Methods

### Subjects

Twenty male participants with ASD and twenty typically developed (TD) male controls were recruited. Participants with ASD were recruited from outpatient units of Karasuyama Hospital, Tokyo, Japan. Controls were recruited through advertising and acquaintances. None of the TDs had any severe medical problem or neurological or psychiatric history, and none met the diagnostic criteria for any psychiatric disorder. Age (ASD: 26.75 ± 3.27; Control: 26.00 ± 5.10; [independent *t*-test; *p*-value: *p* = 0.58, *t*-value: *t*_(38)_ = 0.55, effect size: *d* = 0.18]) and handedness (all subjects were right-handed) were matched between ASD and Control. The diagnostic procedure for patients with ASD was the same as in our previous studies^27,28^. Briefly, an experienced psychiatrist and a clinical psychologist independently interviewed the patients (together with their caregivers when available) for approximately three hours regarding their developmental history, present illness, life history, and family history. The diagnosis of ASD was made only when there was a consensus between the psychiatrist and clinical psychologist based on the criteria of the Diagnostic and Statistical Manual of Mental Disorders, Fourth Edition (DSM-IV-TR)^29^.

The Autism Diagnostic Observation Scale (ADOS)^30^ was administered to all ASD participants, and all but one participant satisfied the diagnostic criteria of ASD (a total score of larger than 6). Although one participant failed to reach the cut-off threshold in terms of total ADOS score by one point, he was included as ASD because he satisfied the cut-off score for a critical subscale of “communication” (3 ≥ 2). The intelligence quotient (IQ) scores of all participants with ASD were evaluated using either the Wechsler Adult Intelligence Scale-Third Edition (WAIS-III) or the WAIS-Revised (WAIS-R) [FIQ: 106.5 ± 12.4, VIQ: 112.0 ± 12.4, PIQ: 98.2 ± 13.3]. Based on the FIQ score, all participants with ASD were considered to be high functioning. On the other hand, IQ scores of Control individuals were estimated using a Japanese version of the National Adult Reading Test [113.13 (mean) ± 6.91 (SD)]^31^. We confirmed that estimated IQs of controls and ASD were well matched (ASD: 110.60 ± 6.61; [independent *t*-test, *p* = 0.24, *t*_(38)_ = −1.18, *d* = −0.37]). In addition, all participants completed the Japanese version of the Autism-Spectrum Quotient (AQ) test^32^ (ASD: 33.8 ± 5.6; Control: 14.7 ± 6.9; [independent *t*-test; *p* = 1.05 × 10^−11^ < 0.01, *t*_(38)_ = 9.60, *d* = 0.48]).

The experiments were approved by the ethics committees of the University of Electro-Communications, the University of Tokyo, and Showa University’s Karasuyama Hospital. All participants received explanations of the experimental procedure and gave their written informed consent. In addition, all procedures were performed in accordance with the Declaration of Helsinki and the guidelines of the Japan Neuroscience Society and the Japan Society of Clinical Neurophysiology.

#### Participants of rs-fMRI dataset

Forty-two adult males with ASD and forty-three age- and gender-matched controls participated in the rs-fMRI scans. Participants with ASD were recruited from outpatient units of Karasuyama Hospital, Tokyo, Japan. Controls were recruited through advertising and acquaintances. The diagnostic procedure for participants with ASD was described previously. None of the controls had any severe medical problem or neurological or psychiatric history, and none met the diagnostic criteria for any psychiatric disorder. The two groups were matched for age (ASD: 29.33 ± 6.74; Control: 27.79 ± 6.37; [independent *t*-test; *p* = 0.28, *t*_(83)_ = 1.09, *d* = 0.24]), handedness (all right-handed), and FIQ (ASD: 109.19 ± 15.15; Control: 109.72 ± 12.80). S2 Table shows the demographic and clinical information of the rs-fMRI dataset.

### Apparatus

Four custom-made force sensors (FSR 402, Interlink Electronics Inc., CA, USA) were used to measure the finger forces in the vertical direction (Fig. 1a). This device was developed and used for our previous study^33^. The circular contact surface of each sensor was covered with a rubber pad. The positions of the force sensors were adjusted in the horizontal plane to match the individual finger positions in which the participants could easily produce finger force. A cushion material was placed under the participant’s palm. The participant’s forearm was fixed to the device with a strap. The force signals were recorded using an AD/DA device (DAQ NI USB-6002, National Instruments, TX, USA) at a 1000-Hz sampling rate. The visual information related to each task was displayed on a laptop computer’s screen (Fig. 1b).

### Task procedures

Participants sat in a chair with their right hand fixed to the finger force measurement device. The measurement experiments consisted of three tasks: (1) a maximal voluntary contraction (MVC) force production task, (2) a single-finger ramp-tracking task for measurement of finger-enslaving (see Fig. S3 for detailed explanation), and (3) a multi-digit force-sharing task. The procedures we adopted were almost the same as those of our previous study^33^. Before the experiments began, the experimenter demonstrated the actual experimental procedure by briefly performing all of the tasks. Details of the MVC force production and a multi-digit force-sharing tasks were as follows.

#### MVC force production task

We measured the MVC force of the four fingers. The participants simultaneously pressed on the force sensors with four fingers and instantaneously produced a maximal total force after a 3-s countdown. The total force was visually fed-back by the height of the vertical bar. This task was repeated twice. We determined the MVC force as the highest value of finger forces.

#### Multi-digit force-sharing task

The participants traced the target path with a visual feedback control and then quickly produced a force pulse in a feedforward manner with all four fingers (Fig. 1b). The small red filled circle was horizontally moved for 8s at a constant speed. The height of the circle corresponded to the total force of the four fingers. The target force template consisted of visual feedback (first 4s) and feedforward (last 4s) control phases. During the feedback-control phase, the participants produced 5% MVC of total force to trace the target path. At the initiation of the feedforward control phase (at the “Go” line), they quickly produced a force pulse within the 25 ± 5% MVC target and immediately relaxed. The circle disappeared at the “Go” line and remained invisible until the end of the feedforward phase. After relaxation, only the peak value of the force pulse was visually fed-back as a stable cursor. We defined successful trials as those in which participants produced a force pulse within the 25 ± 5% MVC target. Participants were informed of success or failure by a message on the screen. The task schedule is shown in Fig. 1d. Participants repeated the task for 200 trials regardless of task success or failure. We changed the weight for the third digit (middle finger) across the blocks to investigate the force-sharing patterns under variable conditions^34^. When the total finger force *F*_*TOT*_ was calculated in real time, the middle finger’s force was weighted by the coefficient *C*. Participants were not informed of this change. They could take a short break between trials to avoid fatigue.

### Rs-fMRI data acquisition

Images were acquired with a 3T MRI scanner MAGNETOM Verio (Siemens Medical Systems, Erlangen, Germany). Functional images were acquired with an echo planar imaging sequence (TR = 2.5 s, TE = 30 ms, flip angle = 80 degrees, matrix = 64 × 64, field of view [FOV] = 212 mm, slice thickness = 3.2 mm, gap = 0.8 mm, 40 axial slices, voxel size = 3.3 × 3.3 × 3.2 mm) at rest for 10 min 10 s. During the scans, participants were instructed to keep looking at a central fixation point, to keep still, to stay awake, and to not think about specific things. T1-weighted anatomical images were acquired for normalization purposes (TR = 2.3 s, TE = 2.98 ms, flip angle = 9 degrees, matrix = 256 × 256, FoV = 256 mm, slice thickness = 1 mm, voxel size = 1 × 1 × 1 mm).

### Behavioral data analysis

To evaluate the morphological 2D:4D ratio, we took digital photos of the participant’s hand as shown in Fig. S5 (we took photos of 20 ASD subjects and 14 TD controls). We defined the finger length as the distance between the center of metacarpophalangeal joint crease and the center of the fingertip on the palmar surface of the hand. We measured the finger length and calculated the 2D:4D ratio by using the number of pixels in the photos.

As a post-hoc validation, we examined the repeatability of the measurement of finger lengths and calculation of the 2D:4D ratio from digital photos. Six persons (one of the authors, S.T., and five persons naïve to the purpose of this study) measured the finger lengths in the 34 photos by using the above method. We calculated the coefficient of variation (CV) for each finger (2D or 4D) length in each photo, which is the ratio of standard deviation to the mean across the lengths measured by the six persons. The CV averaged across the fingers and photos was 0.823% (<1%), and all CVs were less than 3%. We calculated the standard deviation of the six 2D:4D ratios, each of which was measured by different persons, for each photo of the palm. The mean standard deviation across the photos was 0.0105, and all standard deviations were less than 0.03. Therefore, the effect of the measurer was small, and low repeatability of finger length measurement from photos unlikely affected our results.

For the multi-digit force-sharing task, we calculated the tracing error, success rate, and aiming error for each block. All of the measured force data were filtered with a second-order Butterworth low-pass filter with a 10-Hz cutoff frequency. The tracing error was root mean square (RMS) error from the 5%-MVC target during the visual feedback-control phase, i.e., from 1s to 3s after task onset. The success rate was defined as the ratio of the number of successful trials, i.e. the peak value of force pulse was within the 25 ± 5%-MVC target, to the total number of trials. The aiming error was an error between the 25%-MVC target force and the force produced by a participant at a time point when the force peak appeared.

Using the force data in the single-finger ramp-tracking task (Fig. S3), we calculated the enslaving matrix ***E*** that reflects the unintentional finger force produced by the untested fingers when a tested finger produced force^35^. The ratio (*k*) of each finger force (*F*) to the total force (*F*_*TOT*_) was calculated using the data during the middle 12s as follows:

1 $${k}_{i,j}={F}_{i,j}/{F}_{TOT,j},$$

where subscripts *i*, *j* = {*I* (index), *M* (middle), *R* (ring), *L* (little)}, and *j* indicates the tested fingers. *F*_*i*,*j*_ and *F*_*TOT*,*j*_ denote the individual *i*-finger force and the total force, respectively. The average value of *k*_*i*,*j*_ formed the enslaving matric ***E*** as follows:

2 $${\boldsymbol{E}}=[\begin{array}{cccc}{k}_{I,I} & {k}_{I,M} & {k}_{I,R} & {k}_{I,L}\\ {k}_{M,I} & {k}_{M,M} & {k}_{M,R} & {k}_{M,L}\\ {k}_{R,I} & {k}_{R,M} & {k}_{R,R} & {k}_{R,L}\\ {k}_{L,I} & {k}_{L,M} & {k}_{L,R} & {k}_{L,L}\end{array}]\cdot $$

The enslaving index of *j*-finger *EN*_*j*_ was calculated as follows:

3 $$E{N}_{j}=\sum _{j}{k}_{i,j}/3\,(i\ne j).$$

### Rs-fMRI data analysis

Image preprocessing and statistical analyses were processed using SPM8 (http://www.fil.ion.ucl.ac.uk/spm/, Wellcome Department of Cognitive Neurology, University College London, London).

#### Preprocessing

The first four volumes of echo planar images were discarded to accommodate T1 equilibration. The functional images were motion- and slice-time corrected. The mean echo planar images were then co-registered with the native T1 image, spatially normalized into the Montreal Neurological Institute (MNI) stereotactic space with a resolution of 2 × 2 × 2 mm cubic voxels, and then smoothed with an isotropic Gaussian kernel of 6 mm full-width at half-maximum. The anatomical T1 image was segmented to gray matter, white matter (WM), and cerebro-spinal fluid (CSF) for the following denoising process of motion artifacts. To remove motion artifacts, the temporal series of functional images in each voxel was regressed by the following regressors: (a) six motion parameters, (b) squared (a), (c) first-order temporal derivative of (b), (d) squared (c), (e) mean functional images signal over the WM mask, (f) mean functional images signal over the CSF mask, and (g) mean functional images signal over the whole brain^36,37^. Furthermore, the functional image volumes with extraordinary head motion were removed by calculating frame-wise displacement (FD) thresholded by FD > 0.5 mm^37,38^. There was no significant difference in FD values between ASD and Control groups (ASD: 0.18 ± 0.09 mm; Control: 0.17 ± 0.07 mm; [independent *t*-test; *p* = 0.51, *t*_(83)_ = 0.667, *d* = 0.14]). Consequently, there was no significant difference in number of remaining volumes between ASD and Control groups (ASD: 230.10 ± 14.20; Control: 233.80 ± 10.33; [independent *t*-test; *p* = 0.16, *t*_(83)_ = −1.40, *d* = −0.30]). The temporal series in the remaining volumes were band-pass filtered (0.008 < frequency < 0.09 Hz)^39^.

#### Seed regions of interest

We focused on the left primary sensory-motor cortex, SMA, and anterior lobe of the cerebellum, where the hand areas have been frequently reported. Seed regions of interest (ROIs) were defined as spheres with a 7.5-mm radius (Fig. 5a). Based on previous studies, the seed centers were set at (x, y, z) = (−36, −26, 52) in the primary sensory-motor cortex^40^, (2, −2, 53) in SMA^41^, and (16, −51, −19) in the cerebellum^42^ in the MNI coordinates.

#### Functional connectivity analysis

A seed-to-voxel connectivity analysis was conducted across the whole brain for every participant to create a connectivity map. Specifically, we averaged blood-oxygen-level dependent signal time courses across voxels within each seed ROI and then calculated voxel-wise connectivity as Pearson’s correlation coefficient between the averaged time course and every voxel outside the seed ROI in the brain. Correlation coefficients were converted to normally distributed *z* scores using the Fisher transformation.

#### Statistical inference

Individual functional connectivity maps were put into a random effects analysis including individual mean FD values as nuisance effects. In this analysis, we examined group difference using independent two-sample *t*-tests. To identify regions that showed significantly different functional connectivity between ASD and Control groups, clusters were identified with a cluster-forming threshold of *P* < 0.001, uncorrected, and with a peak height threshold of *P* < 0.05, FWE-corrected.

## Supplementary information

Supplementary material

## References

[1] Manning JT, Baron-Cohen S, Wheelwright S, Sanders G (2001). The 2nd to 4th digit ratio and autism. Dev Med Child Neurol.

[2] Hönekopp J (2012). Digit ratio 2D:4D in relation to autism spectrum disorders, empathizing, and systemizing: a quantitative review. Autism Res.

[3] Manning JT, Scutt D, Wilson J, Lewis-Jones DI (1998). The ratio of 2nd to 4th digit length: a predictor of sperm numbers and concentrations of testosterone, luteinizing hormone and oestrogen. Hum Reprod.

[4] Manning JT (2011). Resolving the role of prenatal sex steroids in the development of digit ratio. PNAS.

[5] Lutchmaya S, Baron-Cohen S, Raggatt P, Knickmeyer R, Manning JT (2004). 2nd to 4th digit ratios, fetal testosterone and estradiol. Early Hum Dev.

[6] Hönekopp J, Watson S (2010). Meta-analysis of digit ratio 2D:4D shows greater sex difference in the right hand. Am J Hum Biol.

[7] Baron-Cohen S (2002). The extreme male brain theory of autism. Trends Cogn Sci.

[8] Baron-Cohen S, Knickmeyer RC, Belmonte M (2005). Sex differences in the brain: implications for explaining autism. Science.

[9] Moore JA (1988). Understanding nature - form and function. Am Zool.

[10] Li ZM, Latash ML, Zatsiorsky VM (1998). Force sharing among fingers as a model of the redundancy problem. Exp Brain Res.

[11] Latash ML, Scholz JP, Schöner G (2002). Motor control strategies revealed in the structure of motor variability. Exerc Sport Sci Rev.

[12] Latash, M. L. Movements that are both variable and optimal. *J Hum Kinet* **34**, 5–13, 10.2478%2Fv10078-012-0058-9 (2012). 10.2478/v10078-012-0058-9 PMC3486422 23125821

[13] Olafsdottir H, Yoshida N, Zatsiorsky VM, Latash ML (2005). Anticipatory covariation of finger forces during self-paced and reaction time force production. Neurosci Lett.

[14] van Duinen, H. & Gandevia, S. C. Constraints for control of the human hand. *J Physiol* **589**, 5583–5593, 10.1113%2Fjphysiol.2011.217810 (2011). 10.1113/jphysiol.2011.217810 PMC3249034 21986205

[15] Mosconi, M. W. *et al*. Feedforward and feedback motor control abnormalities implicate cerebellar dysfunctions in autism spectrum disorder. *J Neurosci* **35**, 2015–2025, 10.1523%2FJNEUROSCI.2731-14.2015 (2015). 10.1523/JNEUROSCI.2731-14.2015 PMC4315832 25653359

[16] Thach WT (1998). A role for the cerebellum in learning movement coordination. Neurobiol Learn Mem.

[17] Gillberg C, Billstedt E (2000). Autism and Asperger syndrome: coexistence with other clinical disorders. Acta Psychiatr Scand.

[18] Green D (2002). The severity and nature of motor impairment in Asperger’s syndrome: a comparison with specific developmental disorder of motor function. J Child Psychol Psychiatry.

[19] Mari M, Castiello U, Marks D, Marraffa C, Prior M (2003). The reach-to-grasp movement in children with autism spectrum disorder. Philos Trans R Soc Lond B Biol Sci.

[20] Glazebrook CM, Elliott D, Lyons J (2006). A kinematic analysis of how young adults with and without autism plan and control goal-directed movements. Motor Control.

[21] Fournier KA, Hass CJ, Naik SK, Lodha N, Cauraugh JH (2010). Motor coordination in autism spectrum disorders: a synthesis and meta-analysis. J Autism Dev Disord.

[22] Gowen E, Hamilton A (2013). Motor abilities in autism: a review using a computational context. J Autism Dev Disord.

[23] Miyahara, M. Meta review of systematic and meta analytic reviews on movement differences, effect of movement based interventions, and the underlying neural mechanisms in autism spectrum disorder. *Front Integr Neurosci* **7**, 10.3389/fnint.2013.00016 (2013). 10.3389/fnint.2013.00016 PMC3607787 23532374

[24] Cook JL, Blakemore SJ, Press C (2013). Atypical basic movement kinematics in autism spectrum conditions. Brain.

[25] Pokrywka L, Rachoń D, Suchecka-Rachoń K, Bitel L (2005). The second to fourth digit ratio in elite and non-elite female athletes. Am J Hum Biol.

[26] Wood S (2003). The ratio of second to fourth digit length in azoospermic males undergoing surgical sperm retrieval: predictive value for sperm retrieval and on subsequent fertilization and pregnancy rates in IVF/ICSI cycles. J Androl.

[27] Itahashi T (2014). Altered network topologies and hub organization in adults with autism: a resting-state fMRI study. PLoS One.

[28] Yamada T (2016). Altered functional organization within the insular cortex in adult males with high-functioning autism spectrum disorder: evidence from connectivity-based parcellation. Mol Autism.

[29] American Psychiatric A. Diagnostic and statistical manual of mental disorders: DSM-IV-TR (4th ed., text revision ed.): American Psychiatric Association (2000).

[30] Lord C (2000). The autism diagnostic observation schedule–generic: a standard measure of social and communication deficits associated with the spectrum of autism. J Autism Dev Disord.

[31] Matsuoka K, Uno M, Kasai K, Koyama K, Kim Y (2006). Estimation of premorbid IQ in individuals with Alzheimer’s disease using Japanese ideographic script (Kanji) compound words: Japanese version of National Adult Reading Test. Psychiatry Clin Neurosci.

[32] Wakabayashi A, Baron-Cohen S, Wheelwright S, Tojo Y (2006). The Autism-Spectrum Quotient (AQ) in Japan: A cross-cultural comparison. J Autism Dev Disord.

[33] Togo S, Imamizu H (2016). Anticipatory synergy adjustments reflect individual performance of feedforward force control. Neurosci Lett.

[34] Park J, Zatsiorsky VM, Latash ML (2011). Finger coordination under artificial changes in finger strength feedback: a study using analytical inverse optimization. J Mot Behav.

[35] Zatsiorsky VM, Li ZM, Latash ML (2000). Enslaving effects in multi-finger force production. Exp Brain Res.

[36] Parvizi J, Rangarajan V, Shirer WR, Desai N, Greicius MD (2013). The will to persevere induced by electrical stimulation of the human cingulate gyrus. Neuron.

[37] Yahata N (2016). A small number of abnormal brain connections predicts adult autism spectrum disorder. Nat Commun.

[38] Power JD, Barnes KA, Snyder AZ, Schlaggar BL, Petersen SE (2012). Spurious but systematic correlations in functional connectivity MRI networks arise from subject motion. Neuroimage.

[39] Admon R, Pizzagalli DA (2015). Corticostriatal pathways contribute to the natural time course of positive mood. Nat Commun.

[40] Horovitz SG, Gallea C, Najee-ullah MA, Hallett M (2013). Functional anatomy of writing with the dominant hand. PLoS One.

[41] Chung GH, Han YM, Jeong SH, Jack CR (2005). Functional heterogeneity of the supplementary motor area. AJNR Am J Neuroradiol.

[42] Mostofsky SH (2009). Decreased connectivity and cerebellar activity in autism during motor task performance. Brain.

