# Supplementary material for: Fourth finger dependence of high-functioning autism spectrum disorder in multi-digit force coordination
Source: Sci Rep. 2019 Feb 11;9:1737. doi: 10.1038/s41598-018-38421-6 (PMC6370864; doi:10.1038/s41598-018-38421-6)
Supplement: Supplementary file 1 — Supplementary material [file 41598_2018_38421_MOESM1_ESM.pdf]

Supplementary material

Fourth finger dependence of high-functioning autism spectrum disorder in multi-digit force coordination

Shunta Togo[1, 2, 3*], Takashi Itahashi[4], Ryuichiro Hashimoto[4], Chang Cai[3], Chieko Kanai[4], Nobumasa Kato[4], Hiroshi Imamizu[3, 5]

[1]Graduate School of Informatics and Engineering, The University of Electro-Communications, Tokyo, Japan

[2]Brain Science Inspired Life Support Research Center, The University of Electro-Communications, Tokyo, Japan

[3]Cognitive Mechanisms Laboratories, Advanced Telecommunications Research Institute International, Kyoto, Japan

[4]Medical Institute of Developmental Disabilities Research, Showa University, Tokyo, Japan

[5]Department of Psychology, The University of Tokyo, Japan

*Corresponding author

E-mail: s.togo@uec.ac.jp (ST)

Supplementary Table S1 | A cluster showing a significantly higher functional connectivity with the cerebellar ROI for ASD group than with that for Control group. Peak coordinates are represented in Montreal Neurological Institute coordinates. Cluster size represents the number of 2-mm cubic voxels. BA, Brodmann area.

	BA	Peak coordinates			Cluster Size	*T*-value at peak	*P*-value FWE-corrected at peak
		x	*y*	*z*			
Occipital lobe	17/18	0	-94	-4	149	5.62	0.009

Supplementary Table S2 | Demographic data for participants in rs-fMRI scans.

	**TD (*n* = 43)**			**ASD (*n* = 42)**			**Statistics**	
	Mean	SD	Range	Mean	SD	Range	*df*	*P*-value
Age (years)	27.8	6.4	22-50	29.3	6.7	22-48	83	0.28
Full-scale IQ	109.7	12.8	80-138	109.2	15.2	74-137	65	0.88
Handedness	97.5	5.7	77.8-100	99.1	4.3	75-100	83	0.14
AQ score	17.1	6.1	5-30	33.9	5.8	20-46	81	<0.001
**ADOS**								
Total				11.7	2.4	7-16		
Communication				4.0	1.3	1-6		
Social reciprocity				7.7	1.5	4-10		

Note: WAIS-III or -R was administrated to all participants with ASD, and the IQ score was estimated for all TDs based on JART.

FIQ score was collected from 25 TDs and all participants with ASD.

AQ score was collected from all TDs and 40 participants with ASD.

ADOS score was collected from 38 participants with ASD.

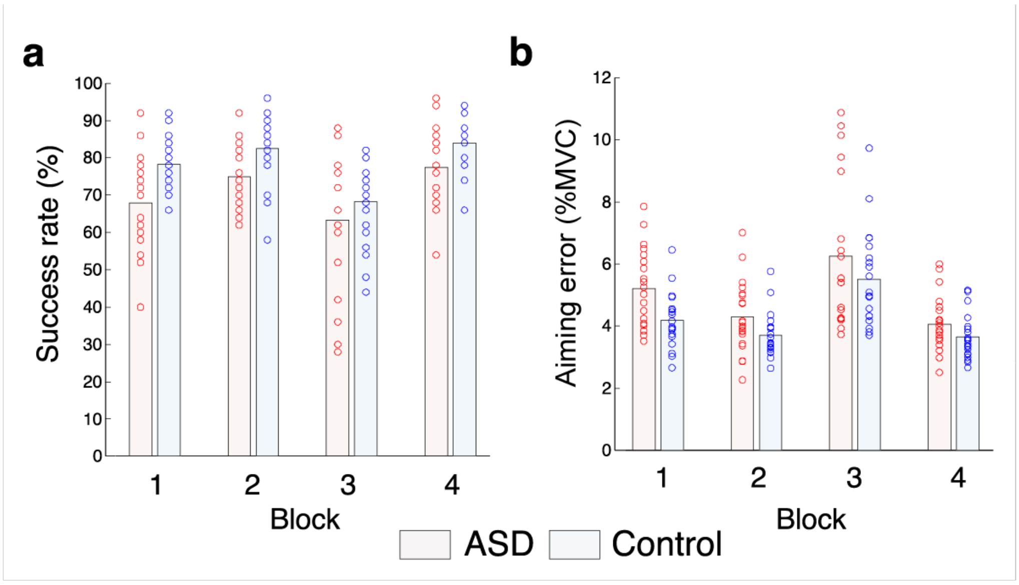

Supplementary Figure S1 | Task performances during feedforward-control phase in each block. (a) Success rate of all participants. Horizontal axis denotes task blocks. Light blue and pink bars denote mean values of ASD and control groups, respectively. Circles correspond to individual participants. (b) Aiming error in feedforward control phase.

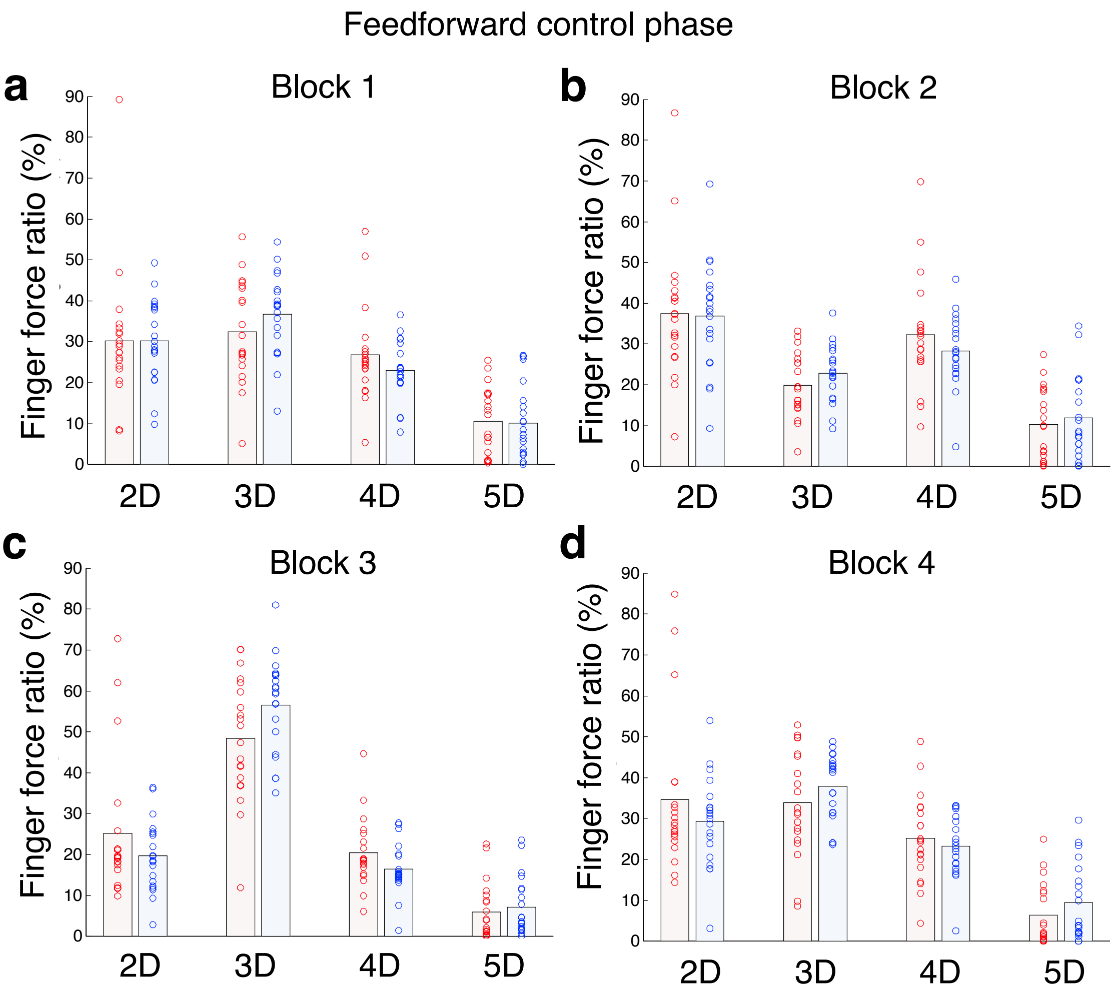

Supplementary Figure S2 | Finger force ratio during feedforward control phase in each block. Finger force ratios from block1 (a) to block4 (d) are shown. The horizontal axis denotes fingers. Light blue and pink bars denote mean values of ASD and control groups, respectively. Circles correspond to individual participants. Finger force of the 3D (middle finger) is differently weighted depending on the task block.

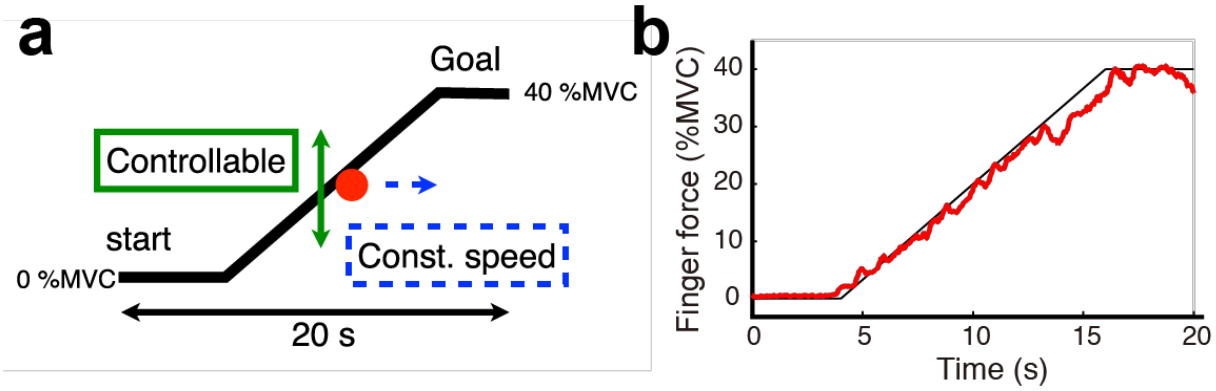

Supplementary Figure S3 | Single-finger ramp-tracking task for measurement of finger enslaving. (a) Screen displayed to participants during the task. A cursor (red circle) moved from left to right for 20 s. Its horizontal speed was constant (2.9 cm/s on the screen), while its vertical position was determined by the finger force of the tested finger. Participants traced the target path (thick black line) with the cursor. They were asked to produce a force only with the tested finger while keeping the rest of their fingers on the sensors. The tested finger was selected from the four fingers (from index to little finger) for every trial. Data of all finger forces were recorded during the task. During the first 4 s, participants kept 0%-MVC force of the tested finger (rest phase). Over the next 12 s, they gradually increased the finger force of the tested finger from 0 to 40% MVC. Finally, they kept 40%-MVC force of the tested finger for 4 s. (b) Typical force trajectory from a participant.

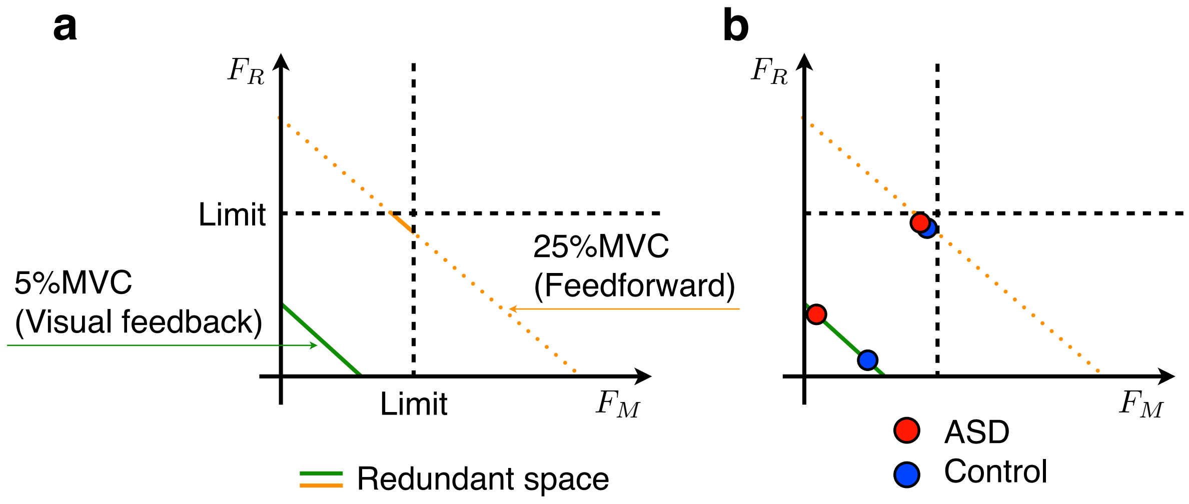

Supplementary Figure S4 | Schematic of the difference in usable redundant space between 5% and 25% MVCs. (a) Horizontal and vertical axes denote the force of middle finger (F_M) and ring finger (F_R), respectively (showing only subspace spanned by two fingers for presentation purposes). Orange and green lines are solution spaces in the cases of 25% MVC and 5% MVC of total force, respectively. When each finger has its limitation of generatable finger force (black dotted line), the usable redundant space when 25% MVC is required (orange solid line) is smaller than that when 5% MVC is required (green solid line). (b) In such a case, the ASD and control participants showed different distributions of finger force when 5% MVC is required. When 25% MVC is required, however, they showed a similar distribution of finger force, since the usable redundant space is small. We assume that the limitation on each finger force under the feedforward-control task is much lower than the maximum voluntarily force of the finger because the task required precise generation of the target force (25% MVC of total force).

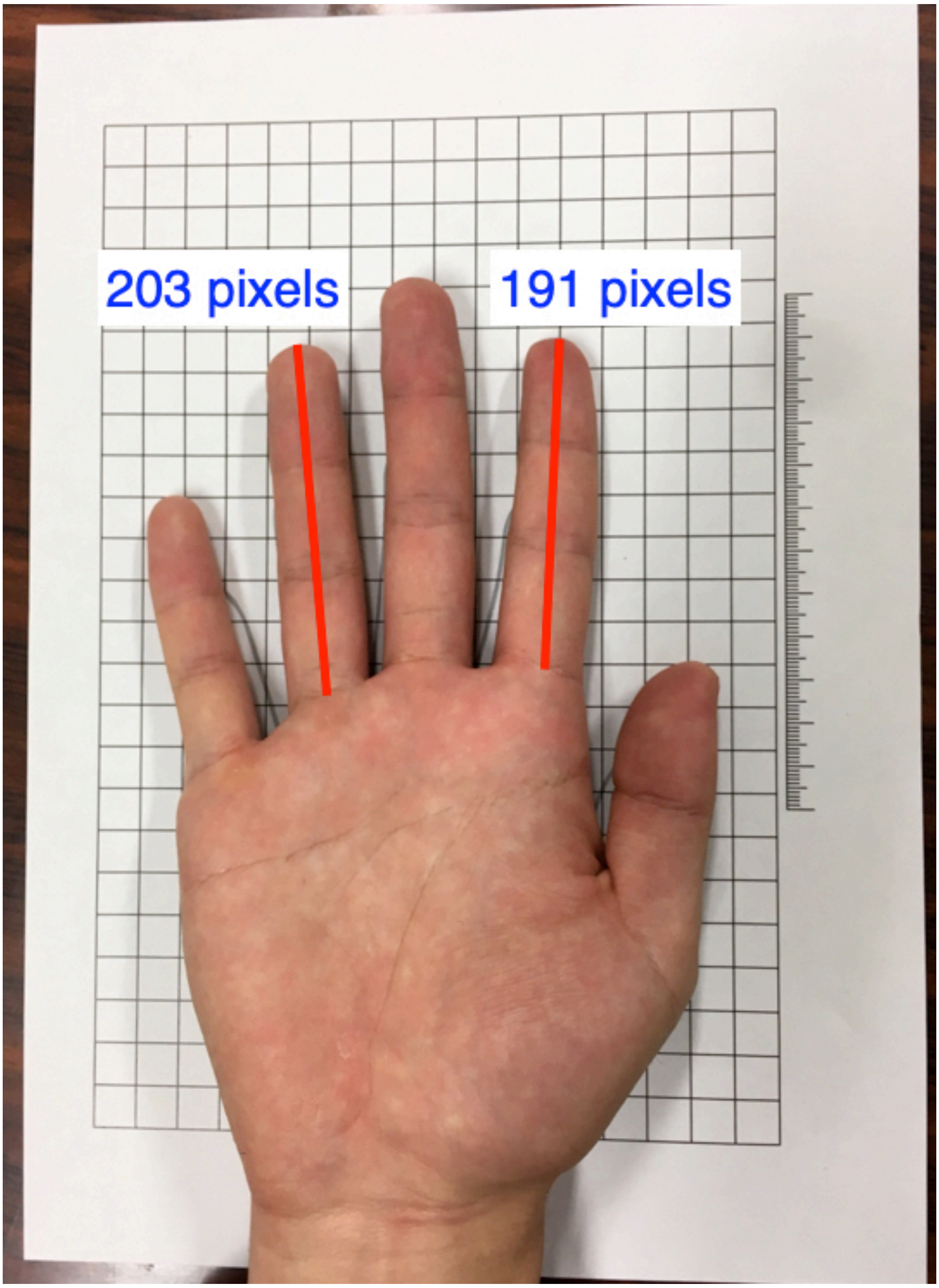

Supplementary Figure S5 | Morphological 2D:4D measurement (photo of a TD control's palm). The number of pixels denotes the distance between the center of metacarpophalangeal joint crease and the center of the fingertip (red lines).
